# Empowering the Future: The Importance of Recognizing Early Career Scientists in *JACC: Basic to Translational Science*

**DOI:** 10.1016/j.jacbts.2024.07.002

**Published:** 2024-08-26

**Authors:** Douglas L. Mann

**Figure:**
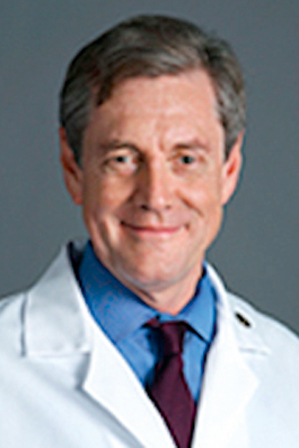

The contributions of early career scientists are indispensable to the field of cardiovascular translational science. These young investigators bring fresh perspectives and incredible energy to our scientific community. The field of cardiovascular translational research, like many scientific disciplines, benefits from diversity and inclusion. Early career scientists, with their varied backgrounds and unique experiences, enrich our research environment. By recognizing the contributions of these diverse voices, *JACC: Basic to Translational Science* promotes a more inclusive scientific community. Simply stated, early career investigators are the future of cardiovascular translational science.

## Fostering Innovation and Creativity

To help foster the careers of early career scientists, *JACC: Basic to Translational Science* has recently begun highlighting early career investigators by publishing their biographical information and their photograph in the Commentary section of the *Journal*, and by promoting them across our various communication channels at the time that their paper is published in the *Journal*. I would like to give a special shout out to Danielle Little (@SodiumDani), who is a senior PhD student in the laboratory of Dr Steven Jones at the University of Louisville, Kentucky. When I met over lunch with the trainees at the University of Louisville and asked them how the *Journal* could help their careers, Danielle suggested that we should provide recognition for early career investigators that went beyond just publishing their papers. The initiative to recognize early career investigators is in direct response to Danielle’s suggestion!

The editors agreed that an early career investigator would be designated as someone who is currently pursuing an advanced degree (PhD or MD or MD/PhD) or who is within 10 years of completing their terminal research degree or the end of their postgraduate clinical training (whichever date is later). Individuals may also request a 2-year eligibility extension for personal reasons. Individuals who are on faculty at the associate professor level would not be regarded as early career investigators.

## Driving the Future of Cardiovascular Research

The editors of *JACC: Basic to Translational Science* recognize that the future of cardiovascular translational research depends on the continued influx of talented new investigators into the field. Accordingly, we are dedicated to promoting the careers of promising early-stage translational scientists by enhancing their visibility within the scientific community whenever possible. As always, we would like to hear your thoughts on this topic, either through social media (*#JACC:BTS;* www.linkedin.com/in/lisamrasmussen) or by email (jaccbts@acc.org).

